# Generation of an Attenuated Chimeric Bat Influenza A Virus Live-Vaccine Prototype

**DOI:** 10.1128/spectrum.01424-22

**Published:** 2022-11-29

**Authors:** Wei Ran, Jacob Schön, Kevin Ciminski, Julian Kraft, Susanne Kessler, Sophie Euchner, Donata Hoffmann, Anne Pohlmann, Martin Beer, Martin Schwemmle, Sebastian Giese

**Affiliations:** a Institute of Virology, University Medical Center Freiburg, Freiburg, Germany; b Faculty of Medicine, University of Freiburg, Freiburg, Germany; c Institute of Diagnostic Virology, Friedrich Loeffler Institute, Federal Research Institute for Animal Health, Greifswald-Insel Riems, Germany; University of Adelaide

**Keywords:** bat influenza A virus, influenza A virus, modified live influenza vaccine

## Abstract

Recurring epizootic influenza A virus (IAV) infections in domestic livestock such as swine and poultry are associated with a substantial economic burden and pose a constant threat to human health. Therefore, universally applicable and safe animal vaccines are urgently needed. We recently demonstrated that a reassortment-incompatible chimeric bat H17N10 virus harboring the A/swan/Germany/R65/2006 (H5N1) surface glycoproteins hemagglutinin (HA) and neuraminidase (NA) can be efficiently used as a modified live influenza vaccine (MLIV). To ensure vaccine safety and, thus, improve the applicability of this novel MLIV for mammalian usage, we performed consecutive passaging in eggs and chickens. Following passaging, we identified mutations in the viral polymerase subunits PB2 (I382S), PB1 (Q694H and I695K), and PA (E141K). Strikingly, recombinant chimeric viruses encoding these mutations showed no growth deficiencies in avian cells but displayed impaired growth in human cells and mice. Homologous prime-boost immunization of mice with one of these avian-adapted chimeric viruses, designated rR65_mono_/H17N10_EP18_, elicited a strong neutralizing antibody response and conferred full protection against lethal highly pathogenic avian influenza virus (HPAIV) H5N1 challenge infection. Importantly, the insertion of the avian-adaptive mutations into the conventional avian-like A/SC35M/1980 (H7N7) and prototypic human A/PR/8/34 (H1N1) viruses led to attenuated viral growth in human cells and mice. Collectively, our data show that the polymerase mutations identified here can be utilized to further improve the safety of our novel H17N10-based MLIV candidates for future mammalian applications.

**IMPORTANCE** Recurring influenza A virus outbreaks in livestock, particularly in swine and chickens, pose a constant threat to humans. Live attenuated influenza vaccines (LAIVs) might be a potent tool to prevent epizootic outbreaks and the resulting human IAV infections; however, LAIVs have several disadvantages, especially in terms of reassortment with circulating IAVs. Notably, the newly identified bat influenza A viruses H17N10 and H18N11 cannot reassort with conventional IAVs. Chimeric bat influenza A viruses encoding surface glycoproteins of conventional IAV subtypes might thus function as safe and applicable modified live influenza vaccines (MLIVs).

## INTRODUCTION

Influenza A viruses (IAVs) originating from aquatic wild-bird reservoirs have recurrently crossed interspecies barriers and established new lineages in various avian and mammalian hosts, including humans (1–3). The ability of avian IAVs to rapidly adapt to new host environments is favored by two distinct mechanisms, antigenic shift, the exchange of genomic information upon coinfection by two parental viruses (reassortment) (4, 5), and antigenic drift, the acquisition of host-specific mutations in single gene segments, which can increase receptor specificity (6, 7), allow immune evasion (8, 9), and bolster viral polymerase activity (10, 11).

The previously discovered bat-derived IAV subtypes H17N10 and H18N11 are not direct descendants of currently circulating avian IAVs (12, 13) but instead exhibit some unprecedented features, presumably as a result of their coevolution with bats. These include altered receptor usage (13, 14) and incompatibility at the RNA and protein levels with conventional IAVs of avian and human origins, preventing reassortment (15–17). Furthermore, bat IAVs lack many of the previously identified mammal-specific amino acid signatures, such as the well-studied PB2_E627K_ adaptation, that are present in many conventional IAVs of human origin and virtually absent in avian strains (18). We and others have previously generated chimeric bat viruses harboring the six so-called internal gene segments (PB2, PB1, PA, NP, M, and NS) of either H17N10 or H18N11 together with segments encoding the hemagglutinin (HA) and neuraminidase (NA) glycoprotein sequences of a conventional IAV strain (15, 16, 19). Due to their inability to reassort with conventional IAVs, these chimeric bat IAVs have emerged as a promising vector backbone for the development of modified live influenza vaccines (MLIVs) in poultry and swine (20–22). However, despite eliciting a potent immune response, the transmission potential and pathogenicity of chimeric bat IAV in ferrets and mice (16, 19, 21) emphasize the need to attenuate these MLIVs prior to future applications in mammals. As recently reported, the attenuation of chimeric bat IAVs can be partially achieved by the truncation of the viral NS1 protein (20). Intriguingly, we previously showed that the passaging of chimeric bat IAVs in eggs and avian cells leads to the rapid selection of various mutations in the PB1, PA, and M gene segments, indicating that the bat IAV backbone has a certain degree of adaptability (15). We therefore speculated that the further passaging of chimeric bat IAV in an avian environment might result in the accumulation of additional mutations in the viral backbone that attenuate viral growth in human cells and mice, thereby increasing the safety of chimeric bat IAV-based MLIVs for mammalian usage.

To explore this in more detail, we generated chimeric bat H17N10 viruses comprising the surface glycoproteins of avian A/swan/Germany/R65/2006 (H5N1) (R65), designated R65_mono_/H17N10, and performed consecutive passaging experiments in eggs and chickens. We identified several additional mutations in the viral polymerase subunits PB2, PB1, and PA of H17N10 that enhanced viral growth in avian cells but resulted in various degrees of attenuation in mammalian cells and mice. Similarly, the insertion of these newly identified mutations into the conventional avian-like A/SC35M/1980 (H7N7) (SC35M) and prototypic human A/PR/8/34 (H1N1) IAVs drastically impaired polymerase activity and viral replication in mammalian cells. Finally, we show that chimeric viruses carrying the identified avian-adaptive mutations can be used as a promising platform to generate safe MLIVs.

## RESULTS

### Passaging of chimeric R65_mono_/H17N10 in embryonated eggs and day-old chicks leads to the selection of adaptive mutations in the polymerase gene segments.

**FIG 1:**
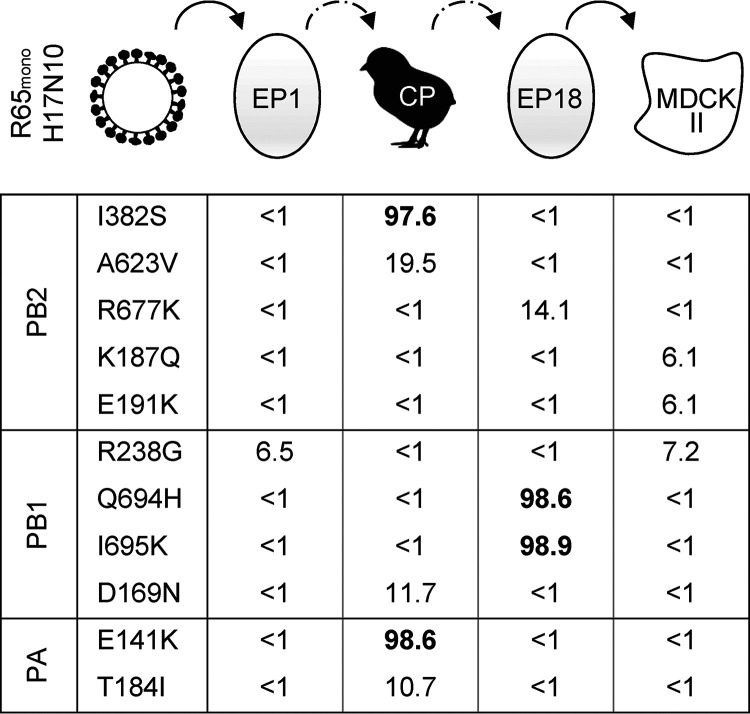
Serial passaging of R65_mono_/H17N10 in eggs and day-old chicks. Recombinant R65_mono_/H17N10 generated on HEK293T cells and subsequently amplified on MDCK II cells was repeatedly passaged (10 times) in 9- to 11-day-old eggs. Virus-containing allantoic fluid was subsequently used to infect day-old chicks (chicken passage [CP]). Virus-positive organ material from the conchae was then consecutively passaged eight times in 11- to 18-day-old eggs (embryonic passage 18 [EP18]). Finally, virus stocks were propagated in MDCK II cells. Deep sequencing of the total vRNA was performed for the passages indicated at the top. Mutation variant frequencies of >90% are highlighted in boldface type.

To identify avian-adaptive mutations that might attenuate viral growth in mammalian cells, we passaged the chimeric bat virus R65_mono_/H17N10 (21) in embryonated eggs of different developmental stages and day-old chicks (see Fig. S1 in the supplemental material). Following egg passage 1 (EP1), EP10, EP12, EP14, and EP18; chicken passage (CP) in day-old chicks; and the final amplification of R65_mono_/H17N10 on Madin-Darby canine kidney II (MDCK II) cells, we isolated viral genomes and performed deep sequencing analyses (Fig. S1). We observed the selection of specific adaptive mutations (≥90% variant frequency) within the viral polymerase proteins PB2, PB1, and PA (Fig. 1 and Fig. S1). In detail, we found (i) the amino acid substitutions I382S in the cap-binding domain of PB2 and E141K in the endonuclease domain of PA and (ii) the substitutions Q694H and I695K within the C-terminal part of PB1 (Fig. S2). The mutations in PB2 and PA emerged simultaneously upon one passage in day-old chicks and gradually disappeared thereafter (Fig. 1 and Fig. S1). In contrast, the two substitutions in PB1 were dominant between EP14 and EP18 but immediately disappeared following one passage in MDCK II cells (Fig. S1). Interestingly, with the exception of E141K in PA, which is found at very low frequencies in avian and human IAV isolates (<0.1%), none of the other substitutions has been detected in any IAV isolate to date (Table S1). Together, these results show that the bat IAV polymerase subunits PB2, PB1, and PA are subject to evolutionary pressure upon the passaging of R65_mono_/H17N10 in embryonated eggs and day-old chicks.

### Amino acid substitutions in the polymerase subunits specifically increase viral replication in avian cells.

**FIG 2:**
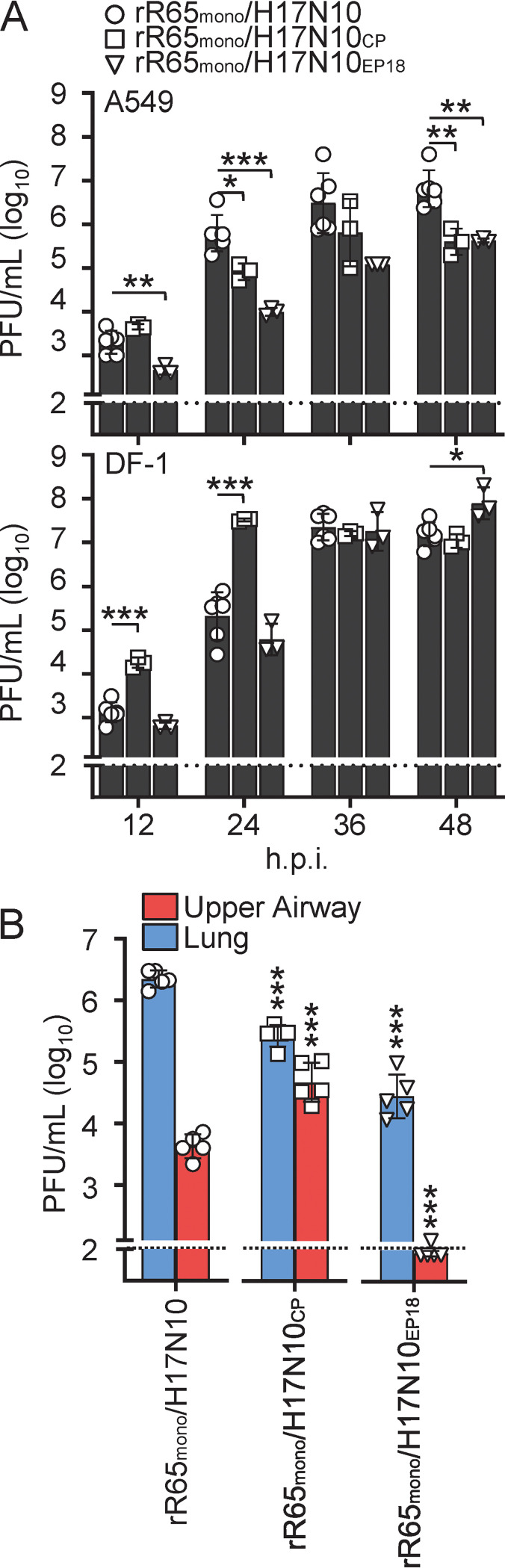
Mutations acquired upon passaging in 18-day-old eggs and day-old chicks impair viral growth in mammalian cells and mice. (A) To determine the impact of the mutations acquired upon passaging in day-old chicks and eggs, we generated recombinant R65_mono_/H17N10 viruses harboring the amino acid substitutions PB2_I382S_ and PA_E141K_ (rR65_mono_/H17N10_CP_) or the two PB1 substitutions Q694H and I695K (rR65_mono_/H17N10_EP18_). Parental rR65_mono_/H17N10 served as the control. Human A549 (top) and avian DF-1 (bottom) cells were infected at a multiplicity of infection (MOI) of 0.01. The virus supernatant was harvested at the indicated time points, and viral titers were determined via a plaque assay. (B) Groups of C57BL/6 mice (*n* = 5) were intranasally infected with 5 × 10^3^ PFU of the indicated viruses in 40 μL, and organ viral titers were determined at 3 dpi via a plaque assay. The dashed lines indicate the detection limit. Data are shown as means ± standard deviations (SD) from ≥3 experiments; statistical analysis was performed using a two-tailed *t* test. *, *P* < 0.05; **, *P* < 0.01; ***, *P* < 0.001.

To determine the impact of the identified polymerase mutations on viral growth, we generated recombinant R65_mono_/H17N10 viruses harboring the CP-selected substitutions PB2_I382S_ and PA_E141K_, designated rR65_mono_/H17N10_CP_, and a virus comprising the two EP18-selected PB1 substitutions Q694H and I695K, which were dominant in EP18, designated rR65_mono_/H17N10_EP18_. Infection of avian (DF-1) and human (A549) cell cultures revealed significantly attenuated viral growth of both rR65_mono_/H17N10_CP_ and rR65_mono_/H17N10_EP18_ in A549 cells compared to wild-type rR65_mono_/H17N10 between 24 and 48 h postinfection (hpi) (Fig. 2A, top) but similar or even higher titers in avian DF-1 cells (Fig. 2A, bottom). Next, we inoculated C57BL/6 mice intranasally with 5 × 10^3^ PFU of rR65_mono_/H17N10, rR65_mono_/H17N10_CP_, or rR65_mono_/H17N10_EP18_ in 40 μL and determined organ viral titers (upper airway and lung) at 3 days postinfection (dpi) (Fig. 2B). rR65_mono_/H17N10 replicated to 5 × 10^3^ PFU/mL in the upper airways and ~10^6^ PFU/mL in the lungs of infected mice (Fig. 2B). In contrast, lung viral titers were 8-fold and 76-fold lower in mice infected with rR65_mono_/H17N10_CP_ and rR65_mono_/H17N10_EP18_, respectively (Fig. 2B). While rR65_mono_/H17N10_CP_ demonstrated enhanced replication properties in the upper airways, the viral titers of rR65_mono_/H17N10_EP18_ were 41-fold lower than those of wild-type rR65_mono_/H17N10. Of note, the increased viral growth of rR65_mono_/H17N10_CP_ in the upper airways was the result of its robust replication properties at cold ambient temperatures (33°C) (Fig. S3). These results indicate that the sequential passaging of R65_mono_/H17N10 in eggs and day-old chicks leads to the transient emergence of avian-adaptive mutations in all viral polymerase subunits, which specifically decrease viral growth in human cells and mice but not avian tissue cultures.

### Avian-adaptive mutations in classical IAV strains cause severe attenuation in mammalian but not avian cells.

**FIG 3:**
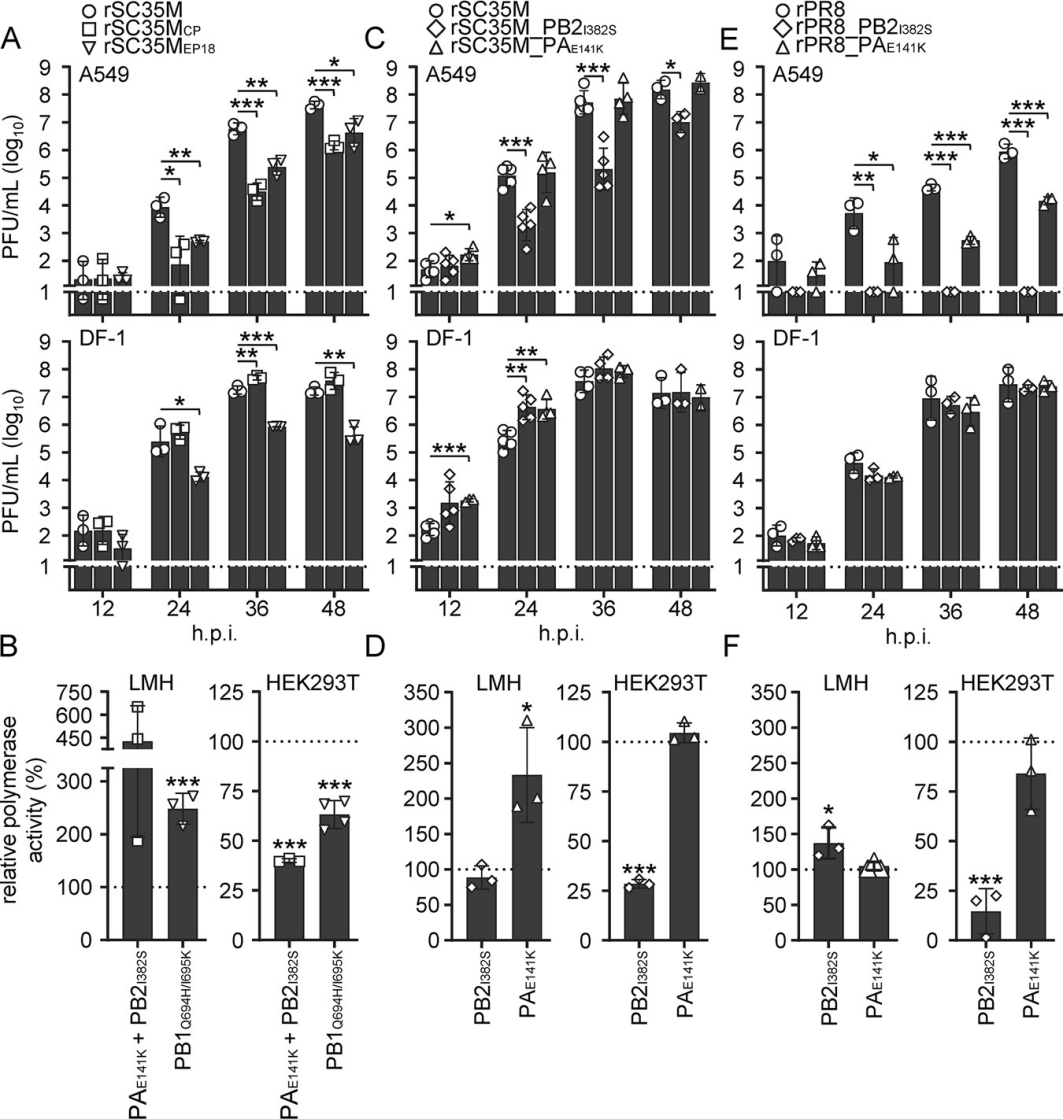
Insertion of the avian-adaptive mutations into A/SC35M/1980 and A/PR/8/34 affects viral growth in human cells. (A) To investigate the mutational impact in the context of conventional IAV isolates, we generated recombinant A/SC35M/1980 viruses comprising either PB2_I382S_ and PA_E141K_ (rSC35M_CP_) or the two PB1 substitutions Q694H and I695K (rSC35M_EP18_). rSC35M served as the wild-type control. Human A549 (top) and avian DF-1 (lower) cell lines were infected at an MOI of 0.001. The virus supernatant was collected at the indicated time points, and viral titers were determined via a plaque assay. The dashed lines indicate the detection limit. (B) Avian LMH and human HEK293T cells were transfected to reconstitute the SC35M polymerase harboring the avian-adaptive mutations PB2_I382S_ and PA_E141K_ or PB1_Q694H/I695K_. At 24 h posttransfection, viral polymerase activity was measured and normalized to the wild-type polymerase activity, which was set to 100% (indicated by the dashed lines). (C and E) The viral growth of SC35M (C) and A/PR/8/34 (E) viruses coding for PB2_I382S_ or PA_E141K_ was evaluated on A549 (top) and DF-1 (bottom) cells infected at an MOI of 0.001. The virus supernatant was collected at the indicated time points, and viral titers were determined via a plaque assay. (D and F) Polymerases of SC35M (D) and PR8 (F) harboring the PB2_I382S_ or PA_E141K_ substitution were reconstituted in LMH and HEK293T cells. At 24 h posttransfection, viral polymerase activity was measured and normalized to the wild-type polymerase activity, which was set to 100% (indicated by the dashed lines). Data are shown as means ± SD from 3 experiments; statistical analysis was performed using a two-tailed *t* test. *, *P* < 0.05; **, *P* < 0.01; ***, *P* < 0.001.

Next, we addressed whether the observed avian-adaptive mutations in PB2, PB1, and PA would cause similar phenotypes in the conventional avian-like A/SC35M/1980 (H7N7) and prototypic human A/PR/8/34 (H1N1) IAV strains. For this purpose, we generated recombinant SC35M viruses encoding PB2_I382S_ together with PA_E141K_, designated rSC35M_CP_, or the two PB1 mutations Q694H and I695K, designated rSC35M_EP18_. Similarly to the replication properties of chimeric rR65_mono_/H17N10_CP_ and rR65_mono_/H17N10_EP18_ (Fig. 2A), rSC35M_CP_ and rSC35M_EP18_ exhibited significantly impaired viral growth in human A549 cells (Fig. 3A, top), demonstrating 29-fold- and 10-fold-lower titers at 48 hpi, respectively. In avian DF-1 cells, rSC35M_CP_, but not rSC35M_EP18_, replicated to higher titers than rSC35M (Fig. 3A, bottom). Given that all amino acid substitutions are located in the viral polymerase proteins, we speculated that they might impair polymerase activity in mammalian cells. We thus reconstituted the SC35M polymerase complex harboring CP (PB2_I382S_ and PA_E141K_) and EP18 (PB1_Q694H/I695K_) signatures and analyzed the luciferase reporter activity in either avian (LMH) or human (HEK293T) cells. As shown in Fig. 3B, the polymerase mutations in the context of SC35M significantly increased viral polymerase activity in avian LMH cells but impaired viral polymerase activity in human HEK293T cells. Interestingly, the single substitution PB2_I382S_ but not PA_E141K_ led to an attenuation of viral growth and affected viral polymerase activity in mammalian but not avian cells (Fig. 3C and D). Next, we tried to recover the corresponding recombinant PR8 viruses, namely, rPR8_CP_, coding for PB2_I382S_ and PA_E141K_, as well as rPR8_EP18_, encoding PB1_Q694H/I695K_, from HEK293T cells or a coculture of HEK293T and LMH cells but repeatedly failed. Consistently, polymerase reconstitution revealed that the insertion of either the CP or EP18 signature rendered the PR8 polymerase nonfunctional in HEK293T cells but had no effect on the activity in LMH cells (Fig. S4). To nonetheless be able to investigate the effect of the mutations identified upon the consecutive passaging of R65_mono_/H17N10 in chicken and eggs, we generated recombinant PR8 viruses harboring either the PB2_I382S_ or PA_E141K_ mutation, designated rPR8_PB2_I382S_ or rPR8_PA_E141K_, respectively. While rPR8_PA_E141K_ was severely attenuated in A549 cells and reached 60-fold-lower titers at 48 hpi than wild-type PR8 (rPR8), the viral growth of rPR8_PB2_I382S_ was completely abrogated in these cells (Fig. 3E, top). In contrast, both rPR8_PB2_I382S_ and rPR8_PA_E141K_ replicated to titers similar to those of rPR8 in DF-1 cells (Fig. 3E, bottom). Upon the reconstitution of the viral polymerases harboring the individual substitution PB2_I382S_ or PA_E141K_, we found that only PB2_I382S_, but not PA_E141K_, severely attenuated the activity of the PR8 polymerase in HEK293T cells (Fig. 3F). As expected, neither of the mutations impaired viral polymerase activity in LMH cells (Fig. 3F).

**FIG 4:**
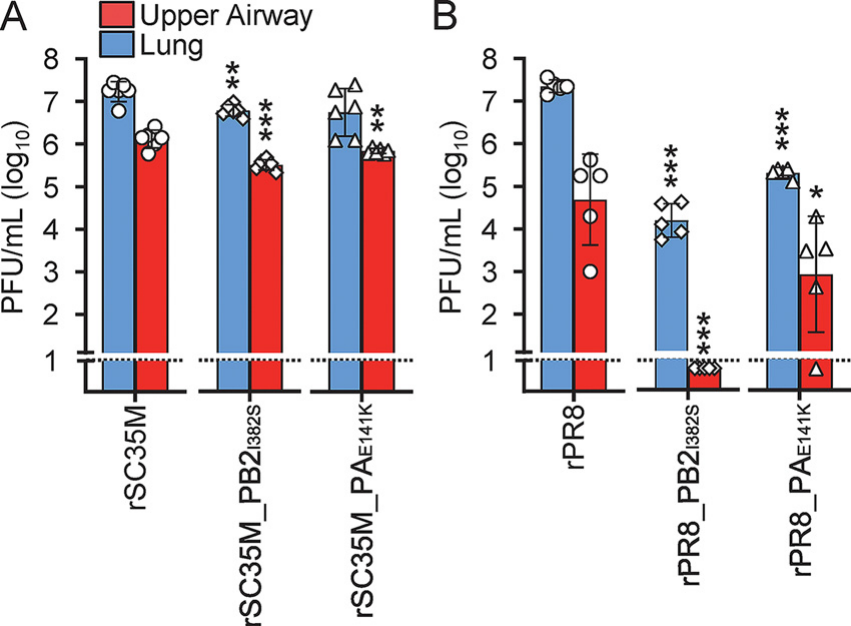
PB2_I382S_ and PA_E141K_ attenuate viral replication of PR8 and SC35M viruses in mice. Groups of C57BL/6 mice (*n* = 5) were intranasally infected with 5 × 10^3^ PFU of SC35M (A) or 5 × 10^2^ PFU of PR8 (B) viruses in 40 μL, and organ viral titers were determined at 3 dpi via a plaque assay. The dashed lines indicate the detection limit. Data are shown as means ± SD statistical analysis was performed using a two-tailed *t* test. *, *P* < 0.05; **, *P* < 0.01; ***, *P* < 0.001.

Upon infection of mice with rSC35M_PB2_I382S_ or rSC35M_PA_E141K_, the organ viral titers were slightly reduced compared to those of wild-type SC35M in the upper airways and, for rSC35M_PB2_I382S_, also in the lungs (Fig. 4A). More strikingly, the viral replication of the prototypic human virus PR8 carrying single avian-adaptive substitutions, rPR8_PB2_I382S_ and rPR8_PA_E141K_, was dramatically attenuated compared to that of wild-type PR8. rPR8_PB2_I382S_ and rPR8_PA_E141K_ replicated to 1,505- and 115-fold-lower titers in the lungs and 4,864-fold- and 122-fold-lower titers in the upper airways, respectively (Fig. 4B). Overall, these findings suggest that the avian-adaptive mutations that were identified in the context of the H17N10 backbone can be transferred to the conventional SC35M and PR8 viruses, thereby attenuating viral growth in both mammalian cells and mice.

### Avian-adapted rR65_mono_/H17N10 is a versatile platform to generate an MLIV.

**FIG 5:**
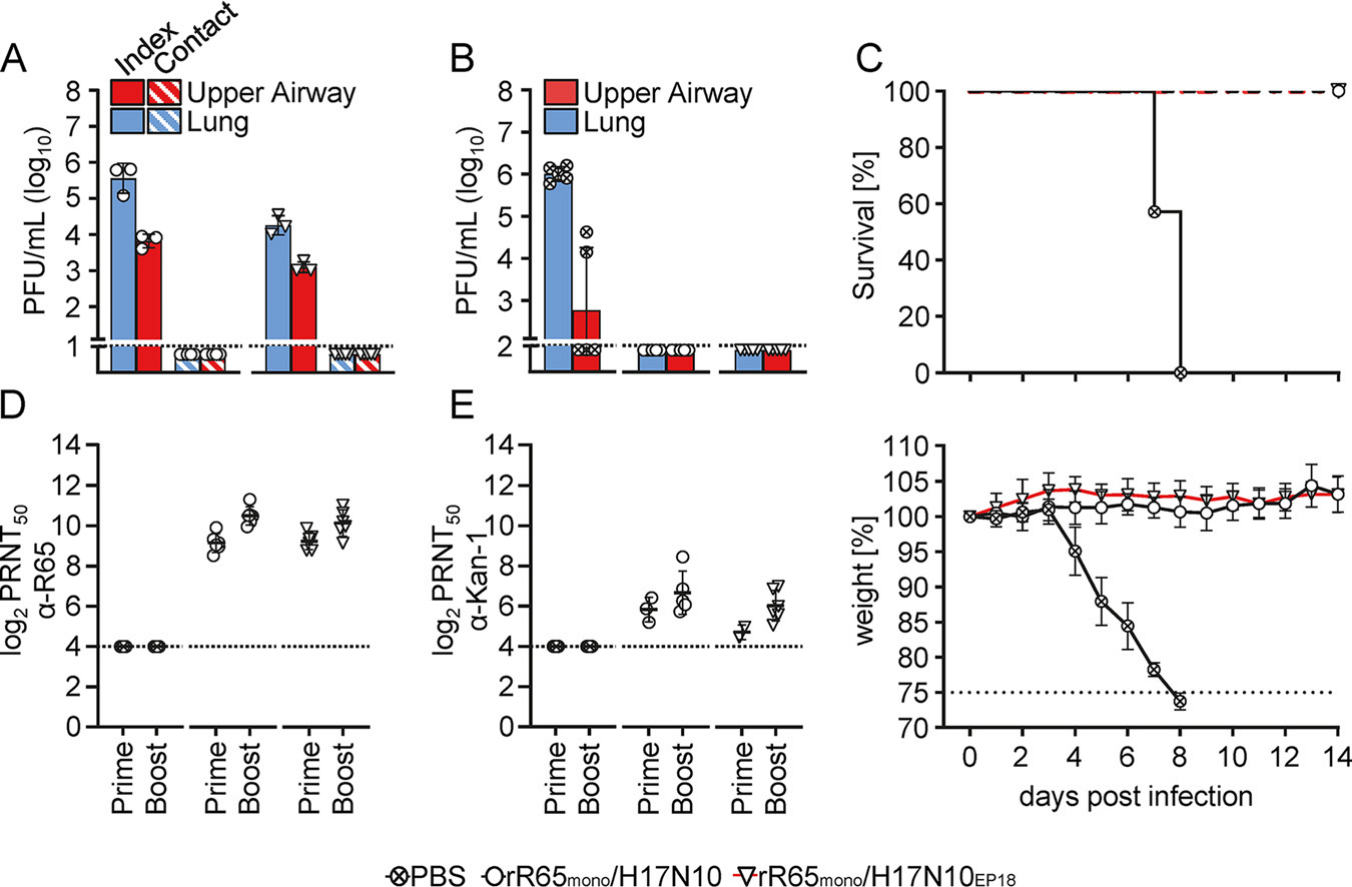
Avian-adapted rR65_mono_/H17N10_EP18_ is a versatile platform to generate MLIVs. (A) To evaluate the transmission potential of the MLIV, groups of C57BL/6 mice (*n* = 3) were inoculated with 5 × 10^3^ PFU of rR65_mono_/H17N10 or rR65_mono_/H17N10_EP18_ in 40 μL intranasally. At 1 dpi, infected index mice were cohoused with naive contact mice (*n *= 5) for 3 days. Organ viral titers were determined at 4 dpi via a plaque assay. MLIV protective efficacies were determined by immunizing groups of mice with 1 × 10^6^ PFU of either rR65_mono_/H17N10 or rR65_mono_/H17N10_EP18_ in 40 μL using a homologous prime-boost vaccine strategy. Mock (PBS)-treated animals served as the controls. Three weeks after the boost, mice were challenged with 3× LD_50_ of A/swan/Germany/R65/2006 (H5N1). (B) Mice of each group (*n *= 5) were sacrificed to determine organ viral titers of the A/swan/Germany/R65/2006 challenge virus at 3 dpi via a plaque assay. (C) Survival and changes in the body weights of the groups (*n* = 7) were monitored for 14 days. Mice were sacrificed and scored dead when 75% of their initial body weight was reached (dashed line). (D and E) Neutralizing antibodies against A/swan/Germany/R65/2006 (H5N1) (D) and A/Thailand/1 (Kan-1)/2004 (E) in the sera of vaccinated and unvaccinated mice were analyzed via a PRNT_50_ assay.

Encouraged by the finding of avian-adaptive mutations, particularly PB1_Q694H/I695K_, that severely attenuate viral replication, we sought to determine whether rR65_mono_/H17N10_EP18_ could be used as a safe platform for future MLIV development. Therefore, we first addressed whether the parental rR65_mono_/H17N10 and the avian-adapted rR65_mono_/H17N10_EP18_ viruses are able to be horizontally transmitted between mice. For this purpose, three mice were intranasally inoculated with 5 × 10^3^ PFU of either virus in 40 μL and then cohoused with five naive contact mice for 3 days. At 4 dpi, we found that intranasal inoculation led to infection of the upper respiratory tract (rR65_mono_/H17N10 = 7 × 10^3^ PFU/mL; rR65_mono_/H17N10_EP18_ = 1.2 × 10^3^ PFU/mL) and lungs (rR65_mono_/H17N10 = 4.6 × 10^5^ PFU/mL; rR65_mono_/H17N10_EP18_ = 2 × 10^4^ PFU/mL) of the index animals. However, we did not observe organ viral titers in either contact group, indicating that none of the viruses were transmitted to the contact animals (Fig. 5A). Moreover, Sanger sequencing of lung homogenates harvested at 4 dpi demonstrated the genetic stability of rR65_mono_/H17N10_EP18_ (Fig. S5). To evaluate the protective efficacy of rR65_mono_/H17N10_EP18_, mice were intranasally immunized with 1 × 10^6^ PFU of the parental rR65_mono_/H17N10 or the avian-adapted rR65_mono_/H17N10_EP18_ virus in 40 μL using a homologous prime-boost vaccine strategy. Three weeks after the boost, mice were given 3× 50% lethal doses (LD_50_) of R65 in 40 μL (3.75 × 10^2^ PFU/mL). Strikingly, at 3 dpi, no infectious virus was detected in either of the vaccinated groups, whereas mice of the mock-treated control group exhibited organ viral titers of ~10^6^ PFU/mL in the lungs and ~10^3^ PFU/mL in the upper respiratory tract (Fig. 5B). While all animals of the mock-vaccinated group succumbed to virus challenge by 8 dpi, all mice of both vaccinated groups survived, without any weight loss (Fig. 5C). We next analyzed neutralizing antibodies in the sera of vaccinated mice via a 50% plaque reduction neutralization titer (PRNT_50_) assay. In line with the absence of infectious virus in the organs of rR65_mono_/H17N10- and rR65_mono_/H17N10_EP18_-vaccinated animals, we determined high levels of neutralizing antibodies after the prime (PRNT_50_ values of 596 for the rR65_mono_/H17N10 group and 629 for rR65_mono_/H17N10_EP18_ group) and even higher titers after the boost (PRNT_50_ values of 1,519 for the rR65_mono_/H17N10 group and 1,240 for the rR65_mono_/H17N10_EP18_ group) (Fig. 5D). Notably, postboost sera also exhibited some degree of heterologous protection against A/Thailand/1 (Kan-1)/2004 (H5N1) (PRNT_50_ values of 116 for the rR65_mono_/H17N10 group and 64 for the rR65_mono_/H17N10_EP18_ group) (Fig. 5E). Together, these data demonstrate that rR65_mono_/H17N10_EP18_ can be used as a MLIV platform, as immunization with this avian-adapted MLIV prototype elicited a humoral immune response and protection against lethal R65 challenge similar to those elicited by vaccination with the more virulent parental rR65_mono_/H17N10 chimera.

## DISCUSSION

Here, we show that the sequential passaging of the chimeric bat R65_mono_/H17N10 virus in embryonated eggs and day-old chicks results in the dynamic adaptation of the viral polymerase subunits PB2 (I382S), PB1 (Q694H and I695K), and PA (E141K). These unique and previously undescribed polymerase mutations led to increased viral growth in avian cells but impaired replication in mammalian cells and mice. In addition, we provide evidence that PB2_I382S_ and PA_E141K_, which appeared following passaging in chicken conchae, allow robust viral replication at lower ambient temperatures. Importantly, the insertion of these mutations into the conventional IAVs SC35M and PR8 similarly affected viral growth in mammalian tissue cultures and mice, implying that these amino acid substitutions prime these strains for replication in an avian environment. While the precise function of the mutations identified here remains to be determined, the cooccurrence of PB2_I382S_ and PA_E141K_ and the localization of these residues within the mRNA cap-binding and the endonuclease domains, respectively, imply that both mutations represent an adaptation to the cap-snatching process. Interestingly, in the context of conventional IAVs, the insertion of PB2_I382S_ alone significantly reduced viral polymerase activity and growth in human cells. In line with this, a recently reported prospective PB2-based deep mutational screening approach identified that residue 382 undergoes positive selection during experimental avian host adaptation (23). Consistently, we propose that the unique properties of chimeric bat influenza A virus-based MLIVs offer new possibilities to study the emergence of avian-adaptive mutations and thereby might enable the identification of as-yet-unknown but critical amino acids required for interspecies transmission. This is best exemplified by the fact that the functions of both PB1 amino acid residues Q694 and I695, which are located in the C-terminal domain, remain obscure.

We further demonstrate that the avian-adaptive mutations Q694H and I695K in PB1 can be utilized to generate a prototypic MLIV backbone that has reduced replication properties in mice compared to the parental virus but induces similarly high levels of protection following immunization. These findings can be used to improve the safety of our previously reported MLIV R65_mono_/H17N10, which provided full protection against lethal H5N1 challenge in both vaccinated chickens and ferrets yet was transmitted to naive contact ferrets during cohousing (21). Lee and colleagues recently presented NS1-truncated chimeric bat MLIVs for the vaccination of pigs (20). Although these NS1-truncated MLIVs were unable to induce sterile immunity against heterologous challenge, they elicited a robust humoral immune response and reduced viral replication. Another recent study reported a cold-adapted MLIV that was protective against homologous challenge in pigs after a single intranasal administration (22). The further development of the bat IAV MLIV backbone and the combination of different attenuating approaches are promising as MLIVs combine the advantages of classical MLIVs, such as their easy oral administration and their induction of a broad adaptive immune response, including mucosal and T cell-mediated immunity (24), with their inherent inability to reassort with circulating IAVs (25). Furthermore, MLIVs offer the decisive advantage that they can be equipped with any set of HA and NA surface glycoproteins, thus enabling targeted immunization against circulating and newly emerging highly pathogenic avian IAVs. In summary, we show that the H17N10-derived polymerase subunits are subject to evolutionary pressure upon the adaptation of chimeric bat IAVs to an avian environment and that these avian-adaptive mutations can be utilized to attenuate MLIVs for mammalian applications.

## MATERIALS AND METHODS

### Cell lines.

A549 human lung adenocarcinoma cells (ATCC CCL-185), HEK293T human embryonic kidney cells (ATCC CRL-3216), Madin-Darby canine kidney II (MDCK II) cells (catalog number 00062107; Merck), LMH chicken hepatocellular epithelial cells (ATCC CRL-2117), and DF-1 chicken fibroblast cells (ATCC CRL-12203) were maintained in Dulbecco’s modified Eagle’s medium (DMEM) supplemented with 10% fetal calf serum (FCS), 100 U/mL penicillin, and 100 mg/mL streptomycin at 37°C with 5% CO_2_.

### Plasmids and generation of recombinant viruses.

Chimeric HA and NA segments were constructed using the HA- and NA-coding regions of A/swan/Germany/R65/2006 (H5N1) (R65) (GenBank accession numbers DQ464354 [monobasic cleavage site HA_RRRKK351T_] and DQ464355) as described previously (15). Briefly, the chimeric HA segment encodes the central coding region flanked at the 3′- and 5′-terminal ends by the putative *cis*-acting terminal packaging sequences from H17N10 (nucleotides 1 to 131 for the 3′ noncoding region and nucleotides 1621 to 1784 for the 5′ noncoding region). The chimeric NA segment comprises the central coding region flanked by the putative *cis*-acting terminal packaging sequences from H17N10 at the 3′ and 5′ ends (nucleotides 1 to 122 for the 3′ noncoding region and nucleotides 1254 to 1390 for the 5′ noncoding region). To prevent premature protein synthesis, all ATG start codons located within the putative terminal packaging sequences were mutated to AAG. The six internal gene segments (PB2, PB1, PA, NP, M, and NS) were from A/little yellow-shouldered bat/Guatemala/153/2009 (H17N10) (GenBank accession numbers CY103873, CY103874, CY103877, CY103875 [PA_S550R_], CY103880, and CY103879 [M1_D156N_ and M2_N31S;T70A_]). pHW2000 rescue plasmids and pCAGGs expression plasmids encoding the polymerase substitutions PB2_I382S_, PB1_Q694H/I695K_, and PA_E141K_ were generated by two-step assembly PCR. All viruses used in this study were generated by the eight-plasmid reverse-genetics system as previously described (15). Recombinant viruses were plaque purified on MDCK II cells and then used for stock generation. Stock titers were subsequently determined via a plaque assay on MDCK II cells.

### Passaging in eggs and day-old chicks.

For the initial inoculation of 10-day-old embryonated specific-pathogen-free (SPF) eggs (*n *= 3), a virus stock of 8.05 × 10^6^ PFU/mL generated on MDCK II cells was used. The amnioallantoic fluid (AAF) of these eggs was harvested at 96 h postinoculation. Viral replication was confirmed by the presence of virus-induced specific cytopathic effect (CPE). AAF was additionally tested for the absence of bacterial or fungal contamination via infection of MDCK II cells (catalog number RIE1061; Collection of Cell Lines in Veterinary Medicine, FLI, Greifswald-Insel Riems, Germany). The AAFs of CPE-positive and contamination-negative eggs were pooled and served as the inoculum for the next passage. Ten consecutive passages in 9- to 11-day-old embryonated eggs were conducted in this way, with a total number of eggs per passage of ≥3. Next, day-old chicks (*n *= 4) were oronasally infected with the virus from the 10th passage. Chick experiments were performed in compliance with German animal protection law (TierSchG). The state of Mecklenburg-Western Pomerania approved the animal experiments (LALLF MV 7221.3-1-023/16). Organ samples of the brain, conchae, lungs, heart, duodenum, and colon were obtained at 4 days postinoculation (4 dpi). The organ samples were placed into reaction tubes containing 1 mL cell culture medium (Dulbecco’s modified Eagle’s medium supplemented with 10% fetal calf serum) supplemented with 1% penicillin-streptomycin and a stainless steel bead (5-mm diameter) and subsequently homogenized (TissueLyser II; Qiagen, Hilden, Germany). Concha samples from two individuals tested positive via PB1-specific reverse transcription-quantitative PCR (RT-qPCR) analysis, with quantification cycle (*C_q_*) values of 32.4 and 34.9, respectively. In order to reisolate virus from these samples, 200 μL of each homogenized sample was used as the inoculum for 10-day-old embryonated SPF eggs (*n *= 5). The AAF was harvested and screened for the presence of the virus using a standard HA assay with 1% chicken red blood cells. Positive tested samples (*n *= 3) were filtered (0.45-μm filter), pooled, and used as the inoculum for an additional egg passage to generate a proper and clean virus stock. A total of 10^3^ 50% tissue culture infective doses (TCID_50_)/egg of this virus stock was used to inoculate 14-day-old embryonated SPF eggs (*n *= 5). After 96 h, the AAF was harvested and analyzed by RT-qPCR. The sample with the lowest *C_q_* value was used as the inoculum for passaging in further-developed 18-day-old embryonated SPF eggs. Following an incubation time of 48 h, the chorioallantoic membrane (CAM), liver, myocardium, duodenum, cecum, cerebrum, and conchae were harvested; processed; and analyzed as described above. The concha tissue with the lowest *C_q_* value was used for further passage. Together, five consecutive passages in 18-day-old embryonated eggs were conducted in this way. The most positive homogenized concha sample of the last passage served as the inoculum for MDCK II cells. The MDCK II cell supernatant was harvested at 72 h postinfection.

### Deep sequencing.

All AAF and cell culture supernatant samples were extracted using the QIAamp MinElute virus spin kit (Qiagen), while sequencing was done on an Illumina MiSeq platform using MiSeq reagent kit v3 (Illumina), as previously described (15). For sequencing of the viral genome directly from concha tissue, the RNA was first extracted from the homogenized organ sample by using TRIzol LS (Thermo Fisher Scientific, Waltham, MA, USA) and a QIAamp viral RNA (vRNA) minikit (Qiagen, Hilden, Germany). Next, influenza virus genome segments were amplified with influenza virus-specific primers using Invitrogen Superscript III one-step RT-PCR with Platinum *Taq* (Thermo Fisher Scientific, Waltham, MA, USA). The following procedures and sequencing on an Ion Torrent PGM instrument (Thermo Scientific, Waltham, MA USA) were conducted as described previously (26). Raw sequence data were quality trimmed and screened for adapter and primer contamination. Consensus sequences were generated using an iterative assembly-and-mapping approach done with Newbler Genome Sequencer software (v.3.0; Roche, Mannheim, Germany) and the Geneious software suite (v.10.0.9; Biomatters, Auckland, New Zealand).

### Viral growth kinetics.

DF-1 and A549 cells were grown to full confluence in six-well plates. The confluent cell layer was washed with phosphate-buffered saline (PBS) and infected with viruses diluted in DMEM containing 0.2% bovine serum albumin (BSA), 100 U/mL penicillin, and 100 mg/mL streptomycin. For all viruses, with the exception of A/SC35M/1980 (H7N7), tosylsulfonyl phenylalanyl chloromethyl ketone (TPCK)-treated trypsin was added (1 μg/mL for A549 and 0.75 μg/mL for DF-1 cells). Viral titers were determined via a plaque assay on MDCK II cells at the indicated time points.

### Polymerase reconstitution assay.

Polymerase reconstitution was performed in HEK293T or avian LMH cells as previously described (27). Briefly, pCAGGs plasmids reconstituting the viral ribonucleoprotein complex consisting of PB2, PB1, PA (45 ng each), and NP (150 ng); the pPolI-FFLuc-RT plasmid coding for the firefly luciferase viral minigenome under the control of either a human or an avian promoter (HEK293T cells, 25 ng; LMH cells, 100 ng); and the pRL-SV40 plasmid encoding the *Renilla* luciferase (20 ng) were cotransfected (28, 29). At 24 h posttransfection, cells were lysed, and the firefly and *Renilla* luciferase activities were measured by using the dual-luciferase reporter assay system (Promega).

### Mouse infections.

All animal experiments were performed in compliance with German animal protection law (TierSchG). The state of Baden-Wurttemberg (Regierungspräsidium Freiburg) approved the animal experiments (reference numbers 35-9185.81/G-21/067 and 35-9185.81/G-19/57). Six- to eight-week-old female isoflurane-anesthetized C57BL/6 mice were inoculated intranasally with the indicated doses of viruses (5 × 10^3^ PFU for H17N10-based viruses, 5 × 10^2^ PFU for PR8-based viruses, and 5 × 10^3^ PFU for SC35M-based viruses) in 40 μL Opti-MEM containing 0.2% BSA. Organs were homogenized in 1 mL PBS by three subsequent rounds of mechanical treatment for 25 s each at 6.5 ms^−1^. Tissue debris was removed by centrifuging homogenates for 5 min at 5,000 rpm at 4°C, and samples were stored at −80°C until further processing. Viral organ titers were determined by a plaque assay.

### Mouse immunization and lethal challenge.

Six- to eight-week-old female isoflurane-anesthetized C57BL/6 mice were immunized with 1 × 10^6^ PFU of the indicated viruses in 40 μL Opti-MEM containing 0.2% BSA by intranasal inoculation. An unvaccinated control group received PBS. Twenty-one days after prime immunization, mice received a homologous boost immunization with 1 × 10^6^ PFU in 40 μL Opti-MEM containing 0.2% BSA. The control group received PBS. Three weeks after the boost, all mice were challenged with 3× LD_50_ of A/swan/Germany/R65/2006 (H5N1) (3.75 × 10^2^ PFU/mL) in 40 μL Opti-MEM containing 0.2% BSA. Body weight loss and signs of disease in infected mice were monitored daily. Postprime (21 days) and postboost (42 days) sera were collected from isoflurane-anesthetized mice by puncture of the facial vein.

### PRNT_50_ test.

Sera of vaccinated and unvaccinated mice were diluted at ratios of 1:16, 1:32, 1:64, 1:128, 1:256, 1:512, and 1:1,024 in a total volume of 50 μL PBS. For each sample, one negative control was included (PBS without serum). Diluted sera and negative controls were subsequently mixed with 90 PFU of either A/swan/Germany/R65/2006 (H5N1) or A/Thailand/1 (Kan-1)/2004 in 50 μL PBS, resulting in final serum dilution ratios of 1:32, 1:64, 1:128, 1:256, 1:512, 1:1,024, and 1:2,048. Following incubation at room temperature for 1 h, 400 μL PBS was added to each sample, and the mixture was subsequently used to infect MDCK II cells. After 1.5 h of incubation at room temperature, the inoculum was removed, and the cells were overlaid with 0.6% Oxoid agar in a mixture containing DMEM, 20 mM HEPES (pH 7.4), 0.1% NaHCO_3_, 1% BSA, and 0.01% DEAE-dextran. Cells were fixed at 48 hpi using 4% formaldehyde for 30 min and stained with 1% crystal violet upon the removal of the agar overlay. PFU were counted manually. Plaques counted for serum-treated wells were normalized to the average number of plaques for the untreated negative controls. The 50% plaque reduction neutralization titer (PRNT_50_) was calculated using a linear regression model in GraphPad Prism 9 (GraphPad Software).

### Viral protein sequence alignments.

Amino acid sequences of the human IAV PB2, PB1, and PA proteins were downloaded from the NCBI Influenza Research Database (https://www.ncbi.nlm.nih.gov/genomes/FLU/Database/nph-select.cgi?go=database). The following criteria were chosen: viral type A, human or avian host, any country/region, any subtype, and full length only. Identical sequences were collapsed to limit sampling biases. Sequences with internal undetermined regions (represented as stretches of more than five X’s) were removed manually. Protein sequences were aligned using the MAFFT multiple-alignment tool (https://mafft.cbrc.jp/alignment/server/), and the amino acid polymorphism at each position was analyzed using the Influenza Research Database Analyze Sequence Variation tool (https://www.fludb.org/brc/snpAnalysis.spg?method=ShowCleanInputPage&decorator=influenza).

### Representation of three-dimensional protein structures.

The three-dimensional structures of influenza A virus polymerase {A/little yellow-shouldered bat/Guatemala/060/2010 (H17N10) [Protein Data Bank (PDB) accession number 4WSB]} were obtained from the Protein Data Bank (https://www.rcsb.org/) and displayed using PyMOL software (www.pymol.org).
