# Supplementary material for: Generation of an Attenuated Chimeric Bat Influenza A Virus Live-Vaccine Prototype
Source: Microbiol Spectr. 2022 Nov 29;10(6):e01424-22. doi: 10.1128/spectrum.01424-22 (PMC9769755; doi:10.1128/spectrum.01424-22)
Supplement: Supplementary file 1 — Fig. S1 to S5 and Table S1. Download spectrum.01424-22-s0001.pdf, PDF file, 0.6 MB [file spectrum.01424-22-s0001.pdf]

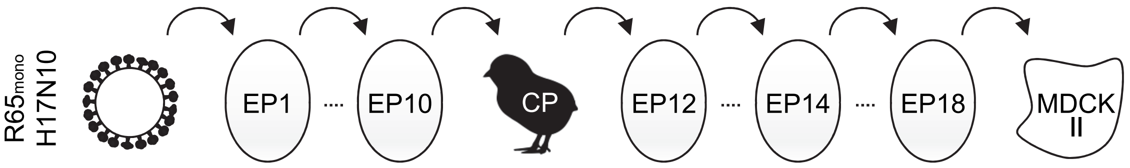

		EP1	EP10	CP	EP12	EP14	EP18	MDCK II
PB2	P456L	<1	11.8	<1	<1	<1	<1	<1
	N472T	<1	10.4	<1	<1	<1	<1	<1
	G377R	<1	14.2	<1	<1	<1	<1	<1
	I382T	<1	8.6	<1	<1	<1	<1	<1
	I382S	<1	<1	**97.6**	**76.2**	<1	<1	<1
	A623V	<1	<1	19.5	<1	<1	<1	<1
	R677K	<1	<1	<1	<1	<1	14.1	<1
	K187Q	<1	<1	<1	<1	<1	<1	6.1
	E191K	<1	<1	<1	<1	<1	<1	6.1
PB1	D48V	<1	8.7	<1	<1	<1	<1	<1
	R238G	6.5	5.6	<1	5.4	<1	<1	7.2
	Q694H	<1	<1	<1	<1	34.9	**98.6**	<1
	I695K	<1	<1	<1	<1	35	**98.9**	<1
	D169N	<1	<1	11.7	<1	<1	<1	<1
	K56Q	<1	<1	<1	<1	38.4	<1	<1
PA	E141K	<1	<1	**98.6**	**75.4**	<1	<1	<1
	T184I	<1	<1	10.7	<1	<1	<1	<1

Supplementary Figure 1: The serial passaging approach of R65$_{mono}$/H17N10 in different avian environments is shown in detail. Recombinant R65$_{mono}$/H17N10 virus underwent consecutive passaging (*n*= 10) in 9-11 day-old eggs (EP1-EP10). Following egg passaging, the virus inoculum was further passaged in day-old chicks before it was isolated from conchae tissue (CP) and utilized to infect (*n*= 2) 9-11 day old eggs (EP12). Following a passage in 14 day old eggs (*n*= 1), the virus was further passaged (*n*= 5) in 18 day-old eggs (EP14-EP18). Finally, virus stocks were amplified in MDCK II cells. Deep sequencing of the total vRNA was performed from passages indicated on top. Mutation variant frequencies of >50% are highlighted in bold.

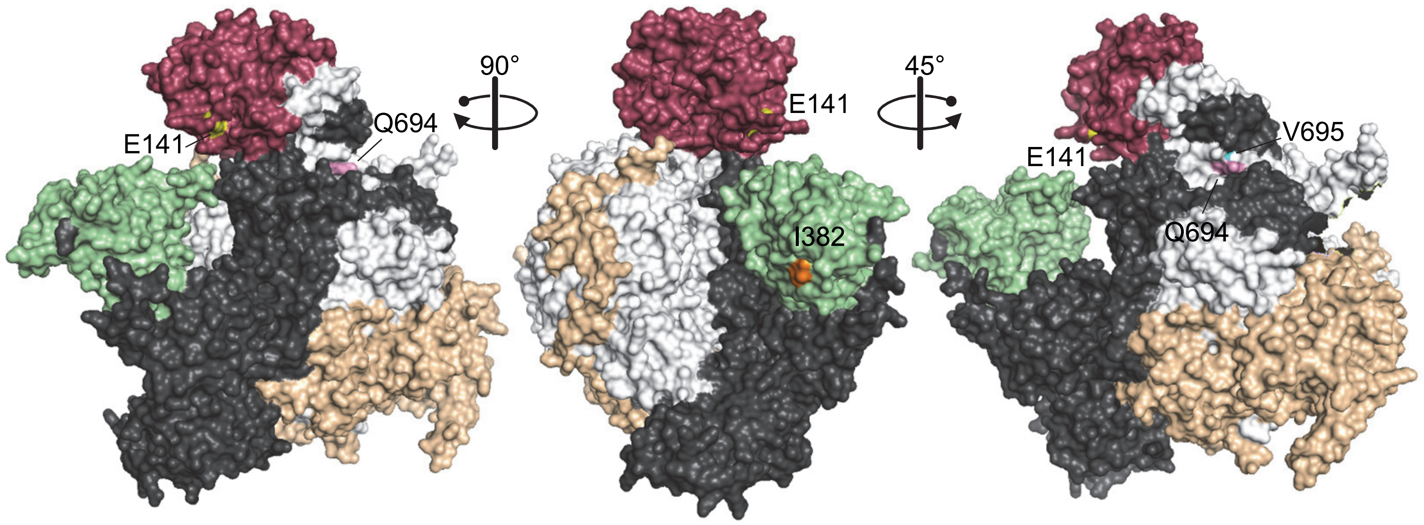

Supplementary Figure 2: Crystal structure of the viral bat polymerase depicting the localization of the avian-adaptive mutations. A cartoon representing the bat IAV H17N10 polymerase was created with PyMol based on the available crystal structure (PDB: 4WSB). The polymerase subunits PA (orange, endonuclease domain is highlighted in red), PB2 (black, cap-binding-domain is highlighted in green) and PB1 (white) are colored for better visibility. The residues undergoing selection in PB2 (I382), PB1 (Q694 and V695) and PA (E141) are indicated.

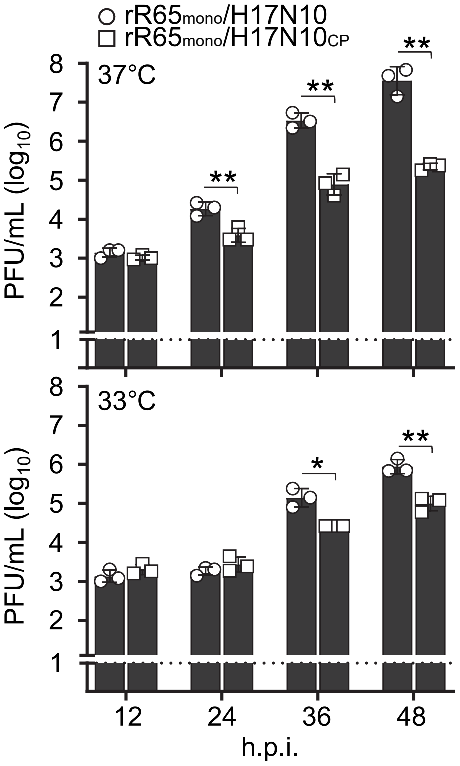

Supplementary Figure 3: rR65$_{mono}$/H17N10$_{CP}$ displays robust replication at cold ambient temperatures. To investigate, whether the amino acid substitutions PB2$_{I382S}$ and PA$_{E141K}$, which were selected following passaging in the chicken conchae, allow for efficient viral replication at cold ambient temperatures, we infected human A549 cells at a MOI of 0.01 and incubated the cells at either 37°C (top panel) or 33°C (lower panel). rR65$_{mono}$/H17N10 served as control. Virus supernatant was harvested at the indicated time points and viral titers were determined via plaque assay. The dashed line indicates the detection limit. Data are shown as mean ± s.d. of *n*=3 experiments; statistical analysis was performed using a two-tailed t-test; * $p< 0.05$; ** $p< 0.01$.

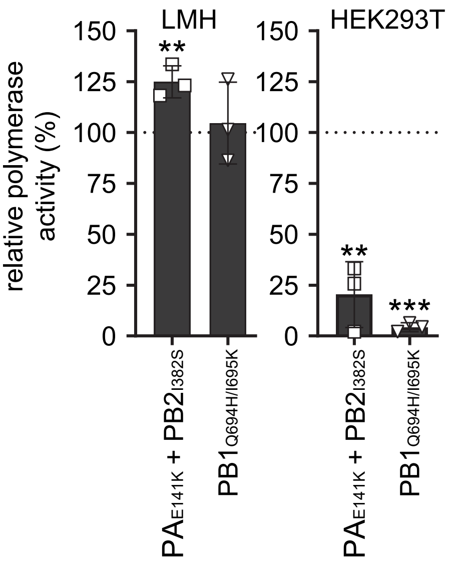

Supplementary Figure 4: Contemporaneous effect of either $PB2_{I382S}$ and PA_{E141K} or the two PB1 substitutions Q694H and I695K on the A/PR/8/34 polymerase. The PR8 polymerase harboring the CP ($PB2_{I382S}$ and PA_{E141K}) or EP18-signature ($PB1_{Q694H/I695K}$) was reconstituted in human HEK293T and avian LMH cells. At 24 hours post transfection viral polymerase activity was measured and normalized to the wild type polymerase activity that was set to 100% (indicated by dashed lines). Data are shown as mean ± s.d. of *n*= 3 experiments; statistical analysis was performed using a two-tailed t-test; * p< 0.05; ** p< 0.01; *** p<0.001.

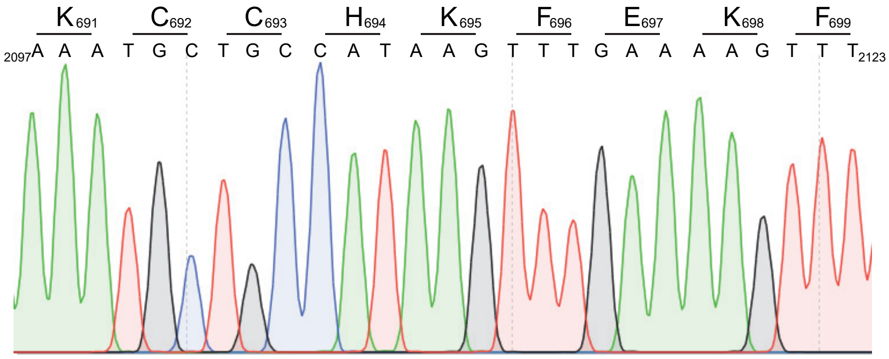

Supplementary Figure 5: The avian-adaptive PB1 mutations Q694H and I695K of rR65$_{mono}$/H17N10$_{EP18}$ are stable in mice.

To demonstrate the stability of the avian-adaptive mutations present within the MLIV candidate rR65$_{mono}$/H17N10$_{EP18,}$ we performed Sanger sequencing of lung homogenates of infected animals at 4 dpi. A representative section of the PB1 sequence covering the inserted mutations (aa: 691-699) is shown.

	Aa 382	Avian (n)	%	Human (n)	%
PB2	I	15.387	98.2	9918	44.3
	V	282	1.8	12.475	55.7
	T	2	-	-	-
	F	-	-	1	-
PB1	Aa 694	Avian (n)	%	Human (n)	%
	N	18.388	87.1	30.967	99.6
	S	2570	12.2	75	0.2
	T	98	0.5	33	0.1
	I	13	0.1	4	-
PB1	Aa 695	Avian (n)	%	Human (n)	%
	L	21.112	99.8	31.097	100
	I	27	0.1	1	-
PA	Aa 141	Avian (n)	%	Human (n)	%
	E	16.482	99.6	22.617	100
	K	17	0.1	3	-
	D	19	0.1	8	-
	G	30	0.2	1	-

Supplementary Table 1: Passaging of chimeric R65$_{mono}$/H17N10 in eggs and day-old chicks results in the selection of unprecedented mutations in the viral polymerase proteins. The avian-adaptive mutations found in the viral polymerase subunits PB2, PB1 and PA are virtually absent in avian or human strains. Analysis was done using MAFFT multiple alignment and "Analyze Sequence Variation" tool.
